# Quality of life and parental stress related to executive functioning, sensory processing, and activities of daily living in children and adolescents with neurodevelopmental disorders

**DOI:** 10.7717/peerj.19326

**Published:** 2025-04-24

**Authors:** Vanesa Lobato-Ruiz, Dulce Romero-Ayuso, Abel Toledano-González, José Matías Triviño-Juárez

**Affiliations:** 1 Department of Occupational Therapy, ASPACE Cáceres, Plasencia, Extremadura, Spain; 2 Department of Physical Therapy (Occupational Therapy Division), Faculty of Health Sciences, University of Granada, Granada, Andalucía, Spain; 3 Mind, Brain and Behavior Research Center (CIMCYC), University of Granada, Granada, Andalucía, Spain; 4 Institute of Biomedical Research IBS, Institute of Biomedical Research IBS, Granada, Andalucía, Spain; 5 University of Castilla-La Mancha, Biomedicine Institute, Albacete, Castilla-La Mancha, Spain; 6 Department of Psychology, Faculty of Health Sciences, Universidad de Castilla La Mancha, Talavera de la Reina, Castilla-La Mancha, Spain; 7 Department of Radiology and Physical Medicine, Faculty of Medicine, University of Granada, Granada, Andalucía, Spain

**Keywords:** Activity of daily living, Children, Executive functions, Neurodevelopmental disorders, Perceived stress, Sensory processing, Quality of life

## Abstract

The quality of life (QoL) of families caring for children with neurodevelopmental disorders is influenced by the severity of the disorder, family support, and access to specialized services. Parental stress also affects family dynamics and QoL due to the additional demands of care, particularly focusing on the management of activities of daily living (ADLs). This study aimed to analyze the relationship between parents’ QoL and stress, involving 46 parents of children aged 3 to 12 years with neurodevelopmental disorders, while also examining the relationship with the performance in ADLs, sensory processing, and executive functioning of children with neurodevelopmental disorders. Significant positive associations were found between factor 1 of the “Assessment of Sensory Processing and Executive Functions in Childhood” (EPYFEI) with perceived stress (*r* = 0.401, *p* ≤ 0.01), and parents physical component summary (PCS) (*r* = 0.330, *p* ≤ 0.05). Significant negative correlations were observed between ADL performance and parents’ physical component summary (PCS) of SF-12 (*r* =  − 0.356, *p* ≤ 0.05). Conversely, a significant negative association was found between factors 1 and 4 of the EPYFEI and ADL performance (*r* =  − 0.392, *p* ≤ 0.01 and *r* =  − 0.660, *p* ≤ 0.01). Furthermore, a significant positive association was found between parents’ perceived stress and the PCS of SF-12 (*r* = 0.471, *p* ≤ 0.01), and a negative association between perceived stress and parents’ mental component summary (MCS) (*r* =  − 0.300, *p* ≤ 0.05). The study revealed that QoL and parental stress are closely linked to functioning in ADLs and executive functioning of children with neurodevelopmental disorders. Interventions to strengthen these areas might improve parents’ well-being and QoL. Additionally, it underscores the importance of teaching these parents stress management strategies.

## INTRODUCTION

Quality of life (QoL) is a multidimensional construct encompassing physical, psychological, social, and environmental aspects, serving as a fundamental concept for evaluating individuals’ well-being (Musetti et al., 2024). It is defined as the subjective perception of an individual’s position in life, considering their cultural context, goals, expectations, and personal concerns (Puka, Conway & Smith, 2020; Romaniuk et al., 2024). QoL is essential to understanding how living conditions affect individuals’ mental and emotional health, and their ability to adapt and face everyday challenges (Bonis, 2016; Vasilopoulou & Nisbet, 2016). The QoL of parents raising children with neurodevelopmental disorders (NDD), such as attention deficit hyperactivity disorder (ADHD), autism spectrum disorder (ASD), and other learning disabilities (American Psychiatric Association, 2014) is significantly affected (Reis et al., 2020). Several factors may be associated with a lower parents’ QoL, including the child’s age, the severity and nature of their manifestations (such as learning and communication difficulties, difficulties in executive functions, differences in sensory processing, and limitations in performing activities of daily living (ADLs)), as well as the presence of comorbidities.

A relevant factor associated with parents’ QoL is their stress level. Specifically, for parents of children with autism, it has been observed that those who use social support and positive reappraisal as coping strategies tend to show a higher QoL in the psychological domain. Conversely, lower QoL is demonstrated by those parents who use aggressive reactions or avoidance as coping strategies. This stress can negatively affect the parents’ QoL, especially when they lack adequate social support to cope with these challenges (Drogomyretska, Fox & Colbert, 2020; Wang et al., 2022). This, along with other parental characteristics (such as gender, perception, coping, stigma and parental self-efficacy), can negatively affect their QoL (Fernández Andrés, Pastor Cerezuela & Botella Pérez, 2016). Other factors that may negatively impact the child’s development include parental characteristics such as their perception of parental self-efficacy, socioeconomic factors (*e.g.*, socioeconomic and employment status) and the level of social and health support available (Craig et al., 2016; Papadopoulos et al., 2023; Papadopoulos et al., 2024). In addition, caring for these children can place a significant emotional and physical burden on parents and family members, negatively affecting the family dynamics (Craig et al., 2016). Parents may experience increased stress due to the additional demands of caregiving, seeking appropriate diagnoses and treatments, and the need to manage multiple aspects of daily life that others do not face, such as the child’s social and behavioral problems, isolation, the experience of bullying, deficits in executive functions (such as planning and organizing their activities), the presence of sensory processing disorders and the need for support to carry out ADLs (Jaffrin, Vinothkumar & George, 2022; Pisula & Porębowicz-Dörsmann, 2017; Reis et al., 2020).

Deficits in executive functions in children with NDD may depend not only on the specific type of disorder but also on the child’s age. Younger children have greater difficulty with inhibitory control, working memory or flexibility, while older children and adolescents experience more difficulty with organizational skills, planning and problem-solving in daily life. In short, this population shows difficulties in self-regulation and in showing goal-directed behaviors (Pardo-Salamanca et al., 2024a; Pardo-Salamanca et al., 2024b). Similarly, difficulties in executive functions have been associated with poorer performance of ADLs. ADLs, which reflect an individual’s ability to function independently, are divided into basic activities (such as personal hygiene and feeding), which are acquired during childhood and become automatic with practice, and instrumental activities, which involve more complex tasks of domestic and social management, developed through education and experience (Gronski & Doherty, 2020). Performing these activities independently is crucial for self-esteem and promotes greater social inclusion and participation in the community. The development of ADLs is a complex process that depends on the individual’s capabilities (physical, motor, cognitive, emotional and psychosocial), the characteristics of the task or activity and the sociocultural context (Yasunaga et al., 2024). In addition to executive functioning, differences in sensory processing in children with ASD and ADHD can affect their daily activities and learning (Sanz-Cervera et al., 2017; Yela-González, Santamaría-Vázquez & Ortiz-Huerta, 2021). Challenges in ADLs experienced by children with NDD can limit their ability to interact socially and affect their QoL (Blanco-Martínez et al., 2020; Lamsal et al., 2020). The inability to perform ADLs not only affects a child’s development but can also have an emotional cost for caregivers and parents, as well as negative consequences for health, social participation, and overall, QoL (Fogel, Rosenblum & Josman, 2020). For children with difficulties in executive functions, these areas can become sources of frustration and persistent obstacles (Barrios-Fernández et al., 2020; Pardo-Salamanca et al., 2024a; Pardo-Salamanca et al., 2024b).

In summary, the QoL of families with children with NDD is profoundly influenced by several interrelated factors, including parental stress, as well as children’s executive functions, sensory processing, and ADL skills (Blanco-Martínez et al., 2020; Frazier et al., 2022; Lamsal et al., 2020). From an intervention perspective, adopting a family-centered approach that considers all these interconnected dynamics is essential (García-Mesa, Delgado-Reyes & Sánchez López, 2021). Developing support programs that address the child’s needs and the family’s emotional well-being is crucial for improving overall QoL (Arias Reyes & Muñoz Quezada, 2019). Interventions should be comprehensive, addressing the various individual and family needs, and include preventive strategies focused on early detection of NDD (Cioni, Inguaggiato & Sgandurra, 2016). Implementing early detection programs in educational and community settings can ensure that children receive timely support, potentially altering their developmental trajectory and improving long-term outcomes (Guralnick, 2017; Zwaigenbaum et al., 2015).

In light of the above, this study aimed to analyze the relationship between executive functions, sensory processing, and ADL performance in children with NDD with parental stress and QoL, as well as the relationship between parental stress and their QoL. Additionally, we aimed to examine the relationship between executive functions and sensory processing in children with NDD and their ADL performance. The hypothesis of this study postulates that greater difficulties in executive functions, sensory processing and lower ADL performance in children with NDD, will be associated with higher levels of stress and lower QoL in parents. Additionally, higher parental stress will be associated with decreased QoL. Moreover, children with NDD with lower executive function and more difficulties in sensory processing will show worse performance in ADL.

## Materials and Methods

### Design

A non-experimental study with a cross-sectional design was conducted.

### Participants

Participants were selected using nonprobability convenience sampling, based on their accessibility and availability. The inclusion criteria were as follows: parents of children aged 3 to 12 years with a diagnosis of NDD, currently receiving treatment at a specialized center; children enrolled in school; have obtained written informed consent from either parents or legal guardians. This age range (3–12 years) was chosen because it represents a period when children with NDD begin facing challenges in academic, social, and family life, which can lead to difficulties in coping and increase the need for greater parental attention and care (Craig et al., 2016). The sample consisted of 43 fathers, 46 mothers, and 46 children from the early intervention and functional habilitation center (ASPACE) in Plasencia, Cáceres, a center dedicated to comprehensive care for children with functional diversity, providing educational, therapeutic, and family support services. The mean age of fathers was 41.37 (4.68) years, and that of mothers was 38.91 (4.25) years. All parents resided in the province of Cáceres. Regarding educational level, the largest proportion of fathers had mandatory education (51.2%), followed by Baccalaureate/Vocational Training (27.9%), university studies (18.6%), and no formal education (2.3%). For mothers, the largest proportion had university studies (37%), followed by mandatory education (28.3%) and Baccalaureate/Vocational Training (28.3%), and lastly, no formal education (6.4%). The main sociodemographic and clinical characteristics of the children are shown in Table 1.

**Table 1:** Sociodemographic characteristics of the child sample (*n* = 46).

Variables		
^1^Age (years) (Mean, SD); (Median; IR)		5.78 (2.60); 5 (4–7.25)
Sex (n, %)	Male	36 (78.3%)
	Female	10 (21.7%)
Term Birth (n, %)	Yes	36 (78.3)
	No	10 (21.7)
Condition^2^ (n, %)	ASD	18 (39.1)
	SLI	14 (30.4)
	DCD	6 (13)
	GDD	8 (17.4%)
Medication (n, %)	Yes	13 (28.3)
	No	33 (71.7)

**Notes.**

1 Variable with non-normal distribution (Shapiro–Wilk test *p*-value < .05); IR: interquartile range.

2 ASD, Autism Spectrum Disorder; SLI, Specific Language Impairment; DCD, Developmental Coordination Disorder; GDD, Global Development Delay.

### Procedure and instruments

The evaluation consisted of administering a questionnaire developed *ad hoc* to collect sociodemographic information from parents (age, sex, educational level, and profession) and sociodemographic (age, sex) and clinical information from children (diagnosis, whether they were receiving pharmacological treatment, pharmacological group), which was completed by the parents. Additionally, the following instruments were administered to parents:

• **Short Form–12 Health Survey (SF-12):** The QoL assessment was conducted using the SF-12 scale adapted for the Spanish population (Schmidt et al., 2012). This questionnaire consists of 12 items that gather information about individuals’ perceived health. Responses were recorded on a Likert scale from one to three or one to five points. The SF-12 identifies eight dimensions grouped into two independent summed scores: Mental Component Summary (MCS) and Physical Component Summary (PCS). The MCS includes the areas of Mental Health (two items), Social Function (one item), Emotional Role (two items), and Vitality (one item), while the PCS covers Physical Function (two items), Physical Role (two items), Bodily Pain (one item), and General Health (one item). Scores range from 0 to 100, the higher the score, the better perceived QoL. The scale has a Cronbach’s alpha of .70 or higher.
• **Perceived Stress Scale (PSS):** This instrument measures the degree to which a person has perceived life as unpredictable, uncontrollable, and overloaded, in addition to the extent to which external demands exceed their perceived capacity to cope (Remor, 2006). This questionnaire has 14 items with a Likert response scale from 0 to 4 (never to very often). Items 1, 2, 3, 8, 11, 12, and 14 pertain to perceived stress, while items 4, 5, 6, 7, 9, 10, and 13 refer to perceived stress coping. The total PSS score is obtained by reversing the scores of items 4, 5, 6, 7, 9, 10, and 13 (in the following way: 0 = 4, 1 = 3, 2 = 2, 3 = 1, and 4 = 0), then summing the 14 items. The higher the score, the higher the level of perceived stress. The questionnaire has a Cronbach’s alpha of .81.
• **Waisman Activities of Daily Living Scale (W-ADL):** The W-ADL scale was used to evaluate the degree of ADL performance (Maenner et al., 2013). This questionnaire has 17 items with three response options, reflecting the ability to perform the activity (0 = cannot do it independently, 1 = can do it with help or support, 2 = can do it independently). Higher scores indicate better performance. The scale has a Cronbach’s alpha of .88–.94.
• **Assessment of Sensory Processing and Executive Functions in Childhood (EPYFEI):** This instrument evaluates Sensory Processing and Executive Functioning, designed for the Spanish population (Romero-Ayuso et al., 2018). It has 34 items divided into five factors: Factor 1 (executive attention, working memory and initiation of actions); Factor 2 (general sensory processing); Factor 3 (emotional and behavioral self-regulation); Factor 4 (supervision, correction of actions, and problem solving); Factor 5 (inhibitory control) with Likert responses from 0 to 4 (0 = never, 1 = almost never, 2 = sometimes, 3 = almost always, 4 = always). A total score > 46.5 points suggests that the child may have a neurodevelopmental disorder and requires further assessment. The scale has a Cronbach’s alpha of .70–.95.

### Statistical analysis

Data analysis was conducted using IBM SPSS Statistics v.29 (IBM SPSS Statistics, Inc., Chicago, IL). The level of statistical significance was set at *p* ≤ 0.05. For descriptive analysis of qualitative variables, absolute frequencies and percentages were used. The Shapiro–Wilk test was performed to check the normality of the distribution of quantitative variables. Mean and standard deviation (SD) or median and interquartile range (IR) were used to describe the quantitative data, depending on whether the distribution was normal (Shapiro–Wilk test *p*-value > 0.05) or not (Shapiro–Wilk test *p*-value ≤ 0.05), respectively. Spearman’s correlation (*r*) was used to explore the association between the main study variables (QoL, perceived stress, ADL functioning, executive functions, and sensory processing) to identify potential relationships between the different constructs studied, considering a weak association when *r* < 0.30, moderate when 0.30 < *r* < 0.70, and strong when *r* > 0.70 (Martínez-González et al., 2020). To ensure data confidentiality, the data analyst did not have access to the data origin or participants’ personal information. A random code was assigned to each participant to guarantee anonymity. Only members of the research team had access to the recorded data.

### Ethical aspects

The research project was approved by the Ethics Committee of Hospital de Cáceres (Ref: 039-2024) and conducted following the ethical principles of the Declaration of Helsinki. Before data collection, written informed consent was obtained from parents or guardians, who were fully informed about the study’s objectives, procedures and risks. There were no risks or direct benefits to the health of the participants.

## Results

According to the EPYFEI scores (Romero-Ayuso et al., 2018), children exhibited difficulties in executive attention, working memory, and initiation of actions (Factor 1). Supervision, correction of actions, and problem solving (Factor 4) were slightly below normal, indicating reduced abilities in these areas. In contrast, scores for general sensory processing (Factor 2), emotional and behavioral self-regulation (Factor 3), and inhibitory control (Factor 5) were within the normal range. The total EPYFEI score was higher than the cut-off, consistent with the diagnosis of NDD. The W-ADL score indicated poor ADL performance (Maenner et al., 2013). The PSS result suggested a moderate level of perceived stress (Remor, 2006). Lastly, the PCS and MCS scores of the SF-12 were below the values for the corresponding age range (Vilagut et al., 2008) (Table 2).

**Table 2:** Descriptive results of executive functioning, sensory processing and performance of ADLs in children, perceived stress and QoL of parents.

Variables	Median (IR)
W-ADL^1^	10 (7–15)
EPYFEI factor 1^1^	23 (9–34)
EPYFEI factor 2^1^	3 (1–7.5)
EPYFEI factor 3^1^	8.5 (5.75–13.25)
EPYFEI factor 4^1^	11.5 (7.75–18.25)
PCS^1^	44.15 (42.67–45.54)
	**Mean (SD)**
EPYFEI factor 5^2^	8.93 (5.03)
EPYFEI total score^2^	58.3 (24.62)
PSS total score^2^	24.4 (9.93)
MCS^2^	45.14 (3.97)

**Notes.**

IR interquartile range W-ADL Waisman Activities of Daily Living Scale EPYFEI Assessment of Sensory Processing and Executive Functions in Childhood PCS Physical Component Summary of the Short Form-12 Health Survey SD Standard deviation PSS Perceived Stress Scale MCS Mental Component Summary of the Short Form-12 Health Survey

1 *p*-value Shapiro–Wilk test ≤ 0.05.

2 *p*-value Shapiro–Wilk test > 0.05.

The analysis of bivariate correlations revealed significant positive and moderate associations between parents’ PSS total score and various factors of the EPYFEI: executive attention, working memory, and initiation of actions (Factor 1), emotional and behavioral self-regulation (Factor 3), supervision, correction of actions, and problem-solving (Factor 4), inhibitory control (Factor 5), and the EPYFEI total score. Additionally, significant positive and moderate associations were found between parents’ PCS and EPYFEI factors 1, 3, and 5, as well as the total score. Furthermore, a significant negative and moderate association was observed between the children’s ADL performance and the parents’ PCS. On the other hand, these analyses showed a significant positive and moderate association between parents’ PSS total score and their PCS, and a significant negative and moderate association between their PSS total score and MCS one. Finally, the results revealed a significant negative and moderate association between EPYFEI factors 1 and 4, as well as the total EPYFEI score, with the children’s ADL performance (W-ADL), and a significant negative and weak correlation between EPYFEI factor 2 and the same performance measure (Table 3).

**Table 3:** Spearman’s correlation between main study variables (*N* = 46).

	EA	SP	ES	Pr-S	IC	EPYFEI	W-ADL	PSS	PCS	MCS
EA	–									
SP	.477^*^	–								
ES	.343^*^	n.s.	–							
Pr-S	.522^**^	.305^*^	n.s.	–						
IC	.351^*^	n.s.	.479^**^	n.s.	–					
EPYFEI	.903^**^	.603^**^	.567^**^	.666^**^	.546^**^	–				
W-ADL	−.392^**^	−.297^*^	n.s.	−.660^**^	n.s.	−.492^**^	–			
PSS	.401^**^	n.s.	.403^**^	.384^**^	.487^**^	.518^**^	n.s.	–		
PCS	.330^*^	n.s.	.461^**^	n.s.	.380^**^	.377^**^	−.356^*^	.471^**^	–	
MCS	n.s.	n.s.	n.s.	n.s.	n.s.	n.s.	n.s.	−.300^*^	n.s.	–

**Notes.**

EPYFEI Assessment of Sensory Processing  and Executive Functions in Childhood Factor 1 (EA) executive attention, working memory and initiation of actions Factor 2 (SP) general sensory processing Factor 3 (ES) emotional and behavioral self-regulation Factor 4 (Pr-S) supervision, correction of actions, and problem solving Factor 5 (IC) inhibitory control W-ADL Waisman Activities of Daily Living Scale PSS Perceived Stress Scale Total score PCS Physical Component Summary of the Short Form-12 Health Survey MCS Mental Component Summary of the Short Form-12 Health Survey n.s. Not Significant

** *p* ≤ 0.01 (bilateral).

* *p* ≤ 0.05 (bilateral).

## Discussion

This study aimed to analyze the relationship between executive functions, sensory processing, and ADL performance in children with NDD with parental stress and QoL, as well as the relationship between parental stress and their QoL. Additionally, we aimed to examine the relationship between executive functions and sensory processing in children with NDD and their ADL performance. The hypothesis of this study suggested that more difficulties in executive functions, sensory processing, and lower ADL performance in children with NDD would be linked to higher stress levels and lower QoL in parents. Additionally, higher parental stress would be associated with lower QoL. Moreover, children with NDD with lower performance of executive function and more sensory processing difficulties would show lower ADL performance.

One of the hypotheses proposed by our study was that lower performance in executive functioning, greater difficulties in sensory processing, and decreased performance in ADLs would be associated with increased parental stress and lower QoL in parents. Pardo-Salamanca et al. (2024a) and Pardo-Salamanca et al. (2024b) reported that significant deficits in executive functions were closely related to greater problems in executing ADLs, and that these were proportional to parental stress, especially when children were unable to successfully carry out ADLs. This suggests the need for specific interventions from occupational therapy that take executive functioning into account to enhance occupational performance in ADLs (Akyurek, Aygun Gurbuz & Irmak, 2024). On the other hand, studies such as that by Fogel, Rosenblum & Josman (2020) indicate that parents of children with (NDD) who experience significant problems in executive functioning face environmental barriers that hinder their autonomy in ADLs and highlight their deficits in executive functioning (Fogel, Rosenblum & Josman, 2020). Additionally, recent studies have shown a relationship between problem-solving ability and perceived difficulty in facing challenges. Chen, Iao & Wu (2024) found that greater problem-solving skills were associated with a lower perception of difficulties when confronting very complex situations (Chen, Iao & Wu, 2024). This may be due to a process of habituation to stress, reducing individuals’ ability to accurately assess the actual level of difficulty they face (Gül & Gür, 2022). Problem-solving ability in children is critical not only for their personal development but also for the daily stress perception and management of their parents. Indeed, an association has been found between children’s competencies in this area, parental stress levels, and the sense of control over the family environment (Higgins et al., 2023). Parents of children with NDD frequently encounter higher stress levels due to the unique and constant demands that their care entails (Ashworth, Palikara & Van Herwegen, 2019). These demands include variables such as specific needs, uncertainty about the child’s development or future, and the challenge of effective communication (Vernhet et al., 2019).

Conversely, it has been found that parents of children with NDD exhibited higher scores in factors such as behavioral problems, emotional issues, and parental stress compared to parents of typically developing children (Operto et al., 2021). Mothers of children with NDD experienced higher levels of anxiety and depression, impacting worse outcomes in coping strategies, positive evaluations, and planning (Megreya et al., 2020). Evidence suggests that mothers experience a greater impact from the difficulties faced by their children compared to fathers, as mothers tend to assume the majority of caregiving responsibilities. This added burden may restrict their professional development and participation in wellbeing, leisure, or self-care activities (Doskalovich, Yochman & Budman, 2025). The difference lies in how fathers and mothers perceive stress regarding their own characteristics and those of the child. This may stem from how they cope with their own challenges and stressors when caring for their child with NDD. In mothers’ cases, it may be due to feeling overwhelmed by the adaptive needs of their child. Fathers may feel stressed due to the societal role of meeting financial needs, which can be exacerbated by the medical and rehabilitation expenses of the child with NDD (Craig et al., 2016). Additionally, increased stress may be related to the parental roles assumed by mothers and fathers within the family unit, exposure to stressors related to the child, or social demands (McStay, Trembath & Dissanayake, 2014; Rivard et al., 2014). Another possible difference in stress perception between parents and mothers may be found in social support, characteristic of the mothers’ stress model. In the work of Pozo & Sarriá (2014), it was shown that a lack of adequate social support to cope with the demands of caring for their child with NDD had a direct and negative effect on mothers’ stress. Conversely, stress in fathers was related to their relationship and attachment, while stress in mothers was related to the demands of childcare (Pozo & Sarriá, 2014). A lack of paternal involvement creates a missed opportunity to identify sources of stress and supports (Johnson & Simpson, 2013).

Regarding the QoL of parents, it has been suggested that the behavioral, emotional, and attentional problems that children face daily directly affect mothers and fathers, significantly reducing their QoL and increasing their emotional, social, and economic demands (Lučić, 2019). Furthermore, a bidirectional association has been found between children’s ADL performance and QoL: if mothers do not feel adequately supported to face their children’s challenges, this may compromise their children’s functioning and wellbeing (Craig et al., 2020). Many parents may lack a stable support system, intensifying their feelings of exhaustion and anxiety. It is noteworthy that some studies have identified that despite perceived stress, parents report high levels of QoL (Bertelli, Francescutti & Brown, 2020; Ritzema et al., 2018), which aligns with our data. These results may be attributable to various factors, such as the strengthening of personal resilience in the face of adversity, the adoption of a more active and healthier lifestyle in response to caregiving demands, or the creation of meaningful social connections through support groups (George et al., 2024). These social networks provide a sense of competence and emotional support, thereby enhancing their overall wellbeing and QoL. Moreover, social support plays a crucial role as a protective factor against diminished QoL, especially for mothers. This is due to a significant association between the ability to receive support from family or social groups and mothers’ capacity to enjoy life (Al-Kandari et al., 2017). Higher levels of social support are associated with lesser negative impact derived from parenting a child with NDD, as well as a reduction in negative mood and depressive symptoms (Lindsey & Barry, 2018). Strong social support improves parents’ mood, translating into better emotional wellbeing and higher QoL. Similarly, when parents feel they have control over events and support from their environment, they are more likely to experience optimism, which predicts positive outcomes in both their QoL and their relationship with their children. This supportive and positive environment fosters better emotional bonds, contributing to the emotional and social development of children and consequently a higher QoL for the family unit (Miranda et al., 2019).

Children with NDD generally face greater difficulties due to their deficits in processing sensory information from their environment. Marco et al. (2011) analyze this evidence, asserting that a deficit in executive attention can increase difficulties in sensory processing (Marco et al., 2011). Furthermore, Greven et al. (2019) argue that the ability to process environmental information is crucial, with differences in this domain potentially leading to cognitive and behavioral difficulties (Greven et al., 2019). Executive functioning and sensory processing are essential for the performance of ADLs. In the case of children with NDD, both factors are often affected. In some instances, these children’s cognitive development levels do not align with their chronological age (Mills, Chapparo & Hinitt, 2020). These data are consistent with our findings, where we identified that deficits in executive functioning and sensory processing are linked to poorer performance in ADLs (Owen et al., 2024; Yela-González, Santamaría-Vázquez & Ortiz-Huerta, 2021). Moreover, the study by Zhang et al. (2023) shows that executive functioning is associated with behavioral factors, emphasizing the importance of considering cognitive interventions for the NDD population based on executive attention and inhibitory control (Zhang et al., 2023). In this sense, Brown, Swayn & Pérez Mármol (2021) also indicate that the association between sensory processing and executive functioning during childhood is essential, finding a significant predictive relationship between sensory processing, measured by the SP-2, and executive functioning measured by the BRIEF-2 (Brown, Swayn & Pérez Mármol, 2021). It has also been demonstrated that these deficits represent an alteration in the regulation of mechanisms at the sensory, motor, and attentional levels (Gandhi & Lee, 2021). The study by Bishara & Kaplan (2022) reinforces the idea that inhibitory control and self-regulation are emotional components associated with ADL performance. These findings align with those of our work, which show a relationship between executive functioning and performance in ADLs (Bishara & Kaplan, 2022) and also support the results of the meta-analysis by Restoy et al. (2024), which highlights the importance of the relationship between emotional regulation and adaptive capacity in children with NDD, which should be considered in interventions (Restoy et al., 2024). The sample analyzed in this study showed a significant correlation between ADL performance and executive functioning. These findings are consistent with other similar studies that have analyzed the importance of executive functioning and the limitations generated by the environment, which are crucial for ensuring autonomy and independence in ADLs (Rosenberg, 2015). In this same vein, Mousavi, Jamali & Raji (2025) found that lower performance in executive functioning corresponded with lower autonomy in ADLs. This underscores the significance of executive functions in the daily challenges faced by children (Mousavi, Jamali & Raji, 2025; Kouklari, Tsermentseli & Monks, 2018).

### Strengths and limitations

This study focuses on a significant topic by analyzing the stress and QoL of parents of children with NDD. It allows us to identify factors associated with parents’ stress and QoL, aspects not frequently explored in previous research. Its understanding is crucial for developing effective support systems tailored to the needs of these families. Additionally, the research employs a clear and structured methodological approach that facilitates replication in subsequent studies, thereby contributing to the validity of its findings. Furthermore, as an exploratory study, it provides useful data that can contribute to more comprehensive future research in this field.

Our study has several limitations that should be considered. The first is related to the absence of a comparison group of neurotypical children and their parents, as well as the study’s cross-sectional design. These prevent determining whether there are differences between these two groups and establishing a causal relationship between the studied variables. Therefore, conducting longitudinal studies with a comparison group could help confirm differences between the groups and whether such a causal relationship exists. Second, the parents and children with NDD were selected using nonprobability convenience sampling, which might introduce selection bias and limit the representativeness of the sample. However, its applicability in exploratory studies like ours has been established (Hernández, Fernández & Baptista, 2014). Third, we did not perform a power analysis to determine the sample size. The small sample size and the heterogeneity in the diagnoses and symptomatology of NDD limit the generalizability of the findings and data interpretation. Increasing the sample size could enable generalisation of the results and perform cluster analyses within subgroups of children with NDD, helping to identify important patterns. Fourth, factors such as the parent’s health status, relevant conditions in other children in the family, and the availability of family or formal support for childcare or household care were not collected. These factors could influence the parents’ QoL. Future studies that include these variables could provide a better understanding of their impact on parental well-being.

## CONCLUSIONS

The study’s results showed that executive functions, sensory processing and ADL performance in children with NDD, were associated with parental QoL and perceived stress. Also, a relationship between parental stress and their QoL was found. Increasing access to resources and support networks for families and developing interventions that address both children’s functional abilities and parents’ psychological well-being are essential. This would not only improve parents’ QoL but also have a lasting and positive impact on the development and well-being of children with NDD (Efstratopoulou et al., 2022).

## Supplemental Information

10.7717/peerj.19326/supp-1 Supplemental Information 1 Raw Data

10.7717/peerj.19326/supp-2 Supplemental Information 2 English-Spanish Codebook

## Additional Information and Declarations

### Field Study Permissions

The following information was supplied relating to field study approvals (i.e., approving body and any reference numbers): ASPACE Cáceres.

### Data Availability

The following information was supplied regarding data availability: The raw data are available in the Supplementary File.
