# Supplementary material for: Quality of life and parental stress related to executive functioning, sensory processing, and activities of daily living in children and adolescents with neurodevelopmental disorders
Source: PeerJ. 2025 Apr 24;13:e19326. doi: 10.7717/peerj.19326 (PMC12034243; doi:10.7717/peerj.19326)
Supplement: Supplemental Information 2 [file peerj-13-19326-s002.docx]

English-Spanish Codebook

Below are translations of the elements that appear in the database for better understanding.

- Código: Code
- Participante: Participant
- Diagnóstico: Diagnosis
- Edad: Age
- Sexo: Sex
- Niño: Biy
- Niña: Girl
- Nivel Escolar: Scholar Level
- TEA: Autism Spectrum Disorder
- TEL: Specific Language Disorder
- TDAH: Attention deficit hyperactivity disorder
- PCI: Childhood Cerebral Palsy
- Educación Infantil: Children Education
- Educación Primaria: Primary Education
- Educación Secundaria: Secundary Education
- Nunca: Never
- Casi nunca: Rarely
- De vez en cuando: Ocasionally
- Ocasionalmente: Sometimes
- A menudo: Often
- Mitad del tiempo: Half Times
- Frecuentemnte: Frecuently
- Muy amenudo: Very Often
- Siempre: Always
- No realiza: Dont Do it
- Con ayuda: With Help
- Independiente: Independent
- Si, me limita mucho: Yes it limits me a lot
- Si, me limita poco, yes it limits me a Little
- No, no me limita nada: no, it doesnt limit me at all
